# Evaluation of the safety, tolerability, pharmacokinetics and pharmacodynamics of SM17 in healthy volunteers: results from pre-clinical models and a first-in-human, randomized, double blinded clinical trial

**DOI:** 10.3389/fimmu.2024.1495540

**Published:** 2024-12-09

**Authors:** Guolin Xu, Sabina Paglialunga, Xuchen Qian, Ru Ding, Kenneth Webster, Aernout van Haarst, Caroline Engel, Chin Wai Hui, Lik Hang Lam, Weimin Li, Wai Chung Wu, Scott Rasmussen, Allen Hunt, Shui-on Leung

**Affiliations:** ^1^ SinoMab BioScience Limited, Hong Kong, Hong Kong SAR, China; ^2^ Celerion Inc., Lincoln, NE, United States

**Keywords:** interleukin-17 receptor B, interleukin-25, alarmins, autoimmune diseases, asthma, humanized antibody

## Abstract

**Background:**

Alarmins mediate type 2 T helper cell (Th2) inflammation and serve as upstream signaling elements in allergic inflammation and autoimmune responses. The alarmin interleukin (IL)-25 binds to a multi-domain receptor consisting of IL-17RA and IL-17RB subunits, resulting in the release of Th2 cytokines IL-4, IL-5, IL-9 and IL-13 to drive an inflammatory response. Therefore, the blockage of IL-17RB via SM17, a novel humanized monoclonal antibody, offers an attractive therapeutic target for Th2-mediated diseases, such as asthma.

**Methods:**

Wild-type mice were stimulated with house dust mite (HDM) extracts for evaluation of SM17’s pre-clinical efficacy in allergic asthma. The safety, pharmacokinectics (PK), pharmacodynamics (PD), and immunogenicity of intravenous (IV) doses of SM17 were assessed in a 2-part clinical study in healthy adult subjects. In Part A, 53 healthy participants were enrolled to receive a single IV dose of SM17 (2, 20, 70, 200, 400, 600, 1200 mg) or placebo. In Part B, 24 healthy subjects were enrolled to receive a single IV dose of SM17 every two weeks (Q2W; 200, 400, 600 mg) or placebo for a total of 3 doses.

**Results:**

Animal studies demonstrated that SM17 significantly suppressed Th2 inflammation in the bronchoalveolar lavage fluid and infiltration of immune cells into the lungs. In the Phase I clinical study, no drug-related serious adverse events were observed. Total SM17 exposure increased by approximately 60- to 188-fold with a 60-fold increase in dose from 20 to 1200 mg SM17. Upon administration of the third dose, mean accumulation ratios over 200-600 mg was 1.5 to 2.1, which confirms moderate accumulation of SM17. After Q2W dosing of SM17 over 4 weeks, total exposure increased in a dose-proportional manner from 200 mg to 600 mg SM17.

**Conclusion:**

In the pre-clinical studies, we demonstrated that SM17 is a potential therapeutic agent to treat allergic asthma. In the Phase 1 clinical trial, a single IV dose of SM17 up to 1200 mg and three Q2W doses up to 600 mg were well tolerated in healthy participants and demonstrated a favorable safety profile. The pre-clinical efficacy and clinical PK and immunogenicity results of SM17 support further clinical development.

**Clinical trial registration:**

https://clinicaltrials.gov/, identifier NCT05332834.

## 1 Introduction

Type 2 T helper cell (Th2) inflammation is mediated by a group of cytokines termed “alarmins”, which include thymic stromal lymphopoietin (TSLP), interleukin (IL)-25 and IL-33. These alarmins serve as upstream signaling elements that are responsible for initiating Th2 immune responses by activating type 2 innate lymphoid cells (ILC2) and Th2 cells, leading to a cascade event resulting in the release of Th2 cytokines (IL-4, IL-5, IL-9, IL-13) and immunoglobulin (Ig)E, which manifests into an allergic inflammatory and autoimmune responses (1, 2).

IL-25 (also referred to as IL-17E) is one of the least studied alarmins, yet was first discovered over 20 years ago (3). It belongs to the IL-17 cytokine family that includes IL-17A to IL-17F. Unlike other members in the family, IL-25 binds to its receptor composed of IL-17 receptor A (IL-17RA) and IL-17 receptor B (IL-17RB) subunits to upregulate transcriptional factors such as NF-KB, STAT6, GATA3 and NF-ATC1, which active and polarize Th2 cells but not Th17 cells, resulting in the expression and secretion of IL-4, IL-5, IL-9 and IL-13 (reviewed in (4)). Upregulation of Th2 cytokines is involved in allergic inflammatory responses in the lungs, epithelial cells and the digestive tract (5, 6). Overall, IL-25 activation results in eosinophilia, in part by delaying eosinophil apoptosis (7), and therefore contributes to eosinophil-driven inflammatory diseases, such as asthma. Moreover, elevated IL-17RB expression was found in lung tissues from asthmatic patients (8), driving the IL-25 effects in this condition.

In asthma, although inhaled corticosteroids (ICS) form the backbone of current therapies, an estimated 24% of patients still exhibit clinical symptoms and are not well-controlled with combinational therapy consisting of ICS and long-acting beta agonists, which could affect patient mortality and quality of life (9). Typical difficult -to-treat asthma is featured with Th2 initiated inflammation (10). Moreover, recent literature suggests that IL-25 was required for induction of allergic airway inflammation. IL-25 concentrations in sputum correlate with disease severity (11), and are associated with type 2 inflammatory response during virus-induced exacerbations (12), as well as with the allergic phenotype (13). As IL-17RB was highly expressed on Th2-related immune cells (Th2 cells, eosinophils, mast cells) (14), and its transcript level in Th2 cells is correlated to serum IgE level during allergic airway inflammation (15), it is not surprising that IL-25 could cooperate with allergen to synergistically induce expressions of MHC-II and co-stimulatory molecules on asthmatic eosinophils, as well as promoting Th2 differentiation of autologous naïve Th2 cells from allergic asthma patients (16). Together with other lung resident cells expressing IL-17RB, it is believed that IL-25 is highly contributed to the inflammation and lung damage via its direct action on lung endothelial cells, antigen presenting cells and Th2-related immune cells during asthma progression (8, 17–19). Therefore, blunting IL-25 activity is an attractive target for asthma treatment. This hypothesis had been tested and validated in experimental animal models of allergic asthma. Antibodies targeting IL-25 could suppress airway hyperresponsiveness (AHR), while the co-treatment with soluble IL-13 receptor α2 (decoy receptor for IL-13) or soluble IL-25 receptor (decoy receptor for IL-25) could synergistically reduce inflammatory cell infiltration, AHR and airway remodeling in allergen induced asthma animal models (20, 21). IL-25 knock-out mice also demonstrated decreased lung pathology during allergen sensitization (22). Moreover, a rare polymorphism in IL-17RB, the signaling receptor for IL-25, is associated with a reduced incidence of asthma (23). Although blockade to the IL-25 pathway is a validated therapeutic approach to asthma in pre-clinical studies, clinical application of targeting the IL-25 pathway is still lacking except a Phase I clinical trial of an IL-25 neutralizing antibody launched in 2023 (NCT05128409) (24).

SM17 is a humanized monoclonal antibody belonging to the IgG4 subtype. Its fragment, antigen binding (Fab) region targets the human IL-17RB. Blockade of IL-17RB by SM17 is expected to interfere with IL-25 signaling, subsequently reducing the Th2-mediated inflammatory response, such as a reduction in cytokine release and eosinophilia in patients. Pre-clinical studies demonstrated that SM17 selectively binds to human IL-17RB, leading to inhibition of alarmin-induced IL-5, IL-9 and IL-13 production, and restoration of Th2 immunity and skin pathology in an animal model of atopic dermatitis (AD) (25). In the current, non-clinical study, the therapeutic role of SM17 in allergic asthma was investigated using a HDM extract-induced mouse model. Lung pathology, inflammatory cell infiltration and cytokine release were all normalized upon SM17 treatment in the HDM model. These encouraging findings support clinical development of SM17 as therapeutic agent for asthma treatment. Therefore, a first-in-human (FIH), randomized, double-blind, placebo-controlled Phase I trial was conducted to evaluate the safety, tolerability, PK, PD and immunogenicity of a single and multiple IV doses of SM17 in healthy participants.

## 2 Materials and methods

### 2.1 SM17 preclinical studies

#### 2.1.1 Animal handling

Wild-type male BALB/c mice (6-8 weeks old) were purchased from the Laboratory Animal Unit, The University of Hong Kong (accredited by the Association for Assessment and Accreditation of Laboratory Animal Care International). All animals were housed in the Specific Pathogen Free (SPF) facility at the Kadoorie Biological Sciences Building at the University of Hong Kong. All animal protocols were approved by the Department of Health in Hong Kong and the Committee on the Use of Live Animals in Teaching and Research (CULATR) of the University.

#### 2.1.2 HDM extract-induced model

HDM extract was used to induce allergic asthma in mice as previously described (26). Briefly, HDM extract (Citeq Biologics, Groningen, NL) was initially reconstituted in filtered saline solution. Anesthetized mice were sensitized by intranasal instillation of 50µg HDM extract on Day 1. The mice were then challenged with 50µg HDM extract for 5 consecutive days starting from Day 8. The pre-clinical antibody formulation of SM17 (either 5 or 2.5 mg/kg), saline control, IgG4 control (5 mg/kg) and dexamethasone (DEX, 1 mg/kg; MCE, Monmouth Junction, NJ) were injected intraperitoneally to designated treatment groups on Day 1, 8, 10 and 12. Mice were euthanized on Day 13 for lung tissue harvest and bronchoalveolar lavage fluid (BALF) collection.

Procedures for BALF collection were mentioned previously (27). Briefly, trachea was exposed and inserted with a catheter into its lumen and tightened by stitches to prevent leakage. One milliliter of BALF collection buffer, containing 100µM ethylenediaminetetraacetic acid (EDTA; ThermoFisher Scientific, Waltham, MA) and 1x Halt™ Protease Inhibitor Cocktail (ThermoFisher Scientific) in phosphate-buffered saline (PBS), was instilled into the lung through the catheter and drawn out immediately. The collected BALFs were centrifuged at 400xg for 7 minutes, and supernatants were saved for determining IL-4, IL-5 and IL-13 concentrations using ELISA kits (R&D systems, Minneapolis, MN) according to the manufacturer’s protocol. Cells in BALF were fixed with 4% paraformaldehyde (PFA; Sigma-Aldrich, Burlington, MA) for identifying ILC2 (recognized as Lineage^-^ CD45^+^ ICOS^+^ ST2^+^) infiltrations with the use of fluorescent antibodies from BioLegend (San Diego, CA).

#### 2.1.3 Lung histology examinations

The lung tissues were fixed in 4% PFA overnight and then dehydrated in ascending concentrations of ethanol. After embedding in paraffin wax, sections of 5μm thickness were prepared with a microtome and mounted on microscope glass slides. Before staining, the sections were deparaffinized in xylene and rehydrated in descending concentrations of ethanol and water. The slides were then stained with hematoxylin and eosin (H&E; Abcam, Cambridge, UK), Periodic acid-Schiff (PAS; Sigma-Aldrich), toluidine blue (Sigma-Aldrich), congo red (Sigma-Aldrich) or Masson’s trichrome (Solarbio, Beijing, CN) for pathological analyses. Lung severity score was assessed by three independent experimenters according to the level of infiltrated cells and bronchiole wall thickness in Image J software (NIH, Bethesda, MD). Data was presented as ± SEM and calculated for statistical significance (p < 0.05) using GraphPad Prism (Version 9, La Jolla, CA).

### 2.2 SM17 clinical studies

#### 2.2.1 Clinical study design

**Figure 1:**
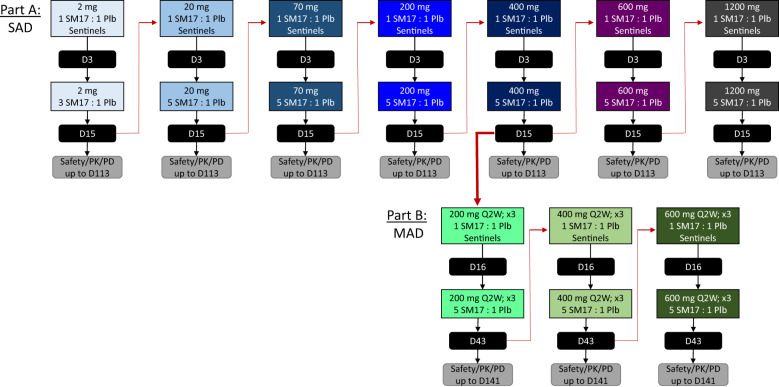
Study Design. Schematic of the single ascending dose (SAD) and multiple ascending dose (MAD) study designs for Part A and Part B, respectively. Black boxes indicate study days the Safety Review Committee convened prior to completing the cohort or initiating the subsequent dose level. D, study day; PD, pharmacodynamics; PK, pharmacokinetic; Plb, placebo; Q2W, every 2 weeks; x3, 3 times for a total of 3 doses.

The FIH study was a 2-part, randomized, double-blind, placebo-controlled trial to evaluate the safety, PK, PD, and immunogenicity of SM17 following single ascending (Part A) and multiple ascending (Part B) IV infusion in healthy adult subjects (ClinicalTrials.gov identifier NCT05332834). Part A included 7 cohorts, where subjects received a single IV infusion of SM17 with doses ranging from 2 mg to 1200 mg or placebo. The first cohort enrolled 6 subjects (4 SM17 and 2 placebo), while the remaining cohorts enrolled 8 subjects (6 SM17 and 2 placebo). In Part B, subjects received a single IV infusion of SM17 or placebo every two weeks (Q2W) over a period of 4 weeks for a total of 3 doses (on Days 1, 15 and 29). The three cohort dose levels explored in Part B were 200, 400 and 600 mg Q2W in 8 subjects each (6 SM17 and 2 placebo). The study employed a sentinel approach to dosing and monitoring, with 1 participant receiving active drug and 1 participant administered placebo, followed by the remaining subjects in each cohort receiving active drug/placebo upon a safety evaluation of the sentinel group by the safety review committee (SRC) (**Figure 1**).

A safety margin of at least 3000-fold was obtained for the starting dose of 2 mg based on the no adverse event level (NOAEL) in mice during a 4-week GLP toxicity study (i.e. 100 mg/kg). The pharmacologically active dose (PAD) was established at 1 mg/kg based on the observed decrease in IL-5 concentrations in BALF in an ovalbumin-induced asthma mouse model (data not shown) and, therefore, the starting dose of 2 mg provided a 30-fold safety margin from the established PAD.

#### 2.2.2 Participants

Healthy, adult participants between 19 to 55 years of age with a body mass index (BMI) of 18 to 32 kg/m^2^ were recruited to participate in the study. Eligible females included postmenopausal women and women of non-childbearing potential; meanwhile, eligible males were instructed to follow contraception guidelines. Participants were excluded if they had a diabetes diagnosis, recently received a live (attenuated) vaccination, had a history of primary immunodeficiency disorder, or received a biologic drug within 90 days of screening. The protocol was reviewed by an Institutional Review Board (Advarra, Columbia, Maryland). All participants provided written informed consent. The trial was conducted at a single center (Celerion, Lincoln, NE) in accordance with the Good Clinical Practice guidelines of the International Conference on Harmonisation and the World Health Organization Declaration of Helsinki.

#### 2.2.3 Study drug

Study drug was supplied as a 200 mg/10 mL solution of SM17 (IgG4 antibody protein) or sterile saline solution for placebo. All doses were administered by IV infusion over 2 hours into a peripheral vein.

#### 2.2.4 Safety evaluation

The primary objective of this trial was to evaluate the safety and tolerability of single and multiple IV doses SM17 in healthy adult subjects. Safety evaluations were assessed throughout the study and included 12-lead safety electrocardiograms (ECGs), vital signs, clinical laboratory tests, infusion site reaction, and physical examinations. Adverse events (AEs) were monitored during the course of the study and coded using MedDRA^®^ Version 25.0. Treatment-emergent adverse events (TEAEs) were defined as any AE occurring after the first study drug administration, or a worsening AE if present at baseline, or was subsequently considered drug-related by the investigator after the first study drug administration.

#### 2.2.5 Sample collection and bioanalytical analysis

Secondary objectives of this trial were to characterize the PK, PD and immunogenicity of single and multiple IV doses of SM17 in healthy adult subjects. In Part A, blood samples to assess serum PK of SM17 were collected prior to start of infusion (SOI), at the end of 2-hour infusion (EOI), then 3, 4, 8, 12, 24 (Day 2), 48 (Day 3), and 72 (Day 4) hours after SOI; then on Day 8, 15, 29, 43, 57, 85, and 113. In Part B, serum samples were obtained for the first dosing interval on Day 1 predose, 2-hour EOI, and at 3, 4, 8, 12, and 24 (Day 2), 48 (Day 3), and 72 (Day 4) hour after SOI, then Day 8; for the second dosing interval on Day 15 at predose, 2-hour EOI, at 3 hours after SOI, then Days 16 and 22; for the third dosing interval, on Day 29 at predose, 2-hour EOI and 3, 4, 8, 12, 24 (Day 30), 48 (Day 31), and 72 (Day 32) hours after SOI, then Days 36, 43, 57, 71, 85, 113, and 141. Serum SM17 concentrations were determined using a validated enzyme linked immunosorbent assay (ELISA) method, with a range of (0.2 – 10.0 µg/mL) (Celerion).

Blood was also collected over the course of the study for total eosinophil and lymphocyte cell counts and phenotyping (Quest Diagnostics, Chantilly, VA). Anti-drug antibodies (ADA) were tested for potential immunogenicity against SM17 using electrochemiluminescence immunoassay (ECLIA) for antibodies, with assay concentrations able to detect ADAs down to 19.1 ng/mL (low positive control) (Celerion).

#### 2.2.6 Statistical analysis

A typical FIH ascending dose study sample size of approximately 8 subjects per cohort was selected, with no formal statistical estimation. Safety parameters were summarized descriptively (SAS for Windows Version 9.4, SAS Institute, Inc., Cary, NC). The primary PK outcomes were serum concentration area-under the curve (AUC) from time zero to Day 14 (AUC_0-14d_), AUC from time zero to the last quantifiable observed non-zero concentration (AUC_0-t_); AUC from time zero extrapolated to infinity (AUC_0-inf_), and AUC during a dosing interval (τ) at steady state (AUC_τ_), as well as maximum observed concentration (C_max_), time to reach C_max_ (T_max_), apparent total serum clearance (CL) and volume of distribution (V). Dose proportionality was determined from evaluation of a powered analysis of the slope estimate and width of the 95% confidence intervals (CI). In both Part A and B, participants administered placebo were pooled for the analysis. A noncompartmental PK approach was used to analyze individual serum SM17 concentration-time data (using Phoenix^®^ WinNonlin^®^ Version 8.3.4; Certara USA, Inc., Princeton, NJ). Graphs were produced using Prism GraphPad Version 7.05.

## 3 Results

### 3.1 Preclinical studies: SM17 ameliorates asthma phenotypes in HDM extract-induced asthma mouse model

**Figure 2:**
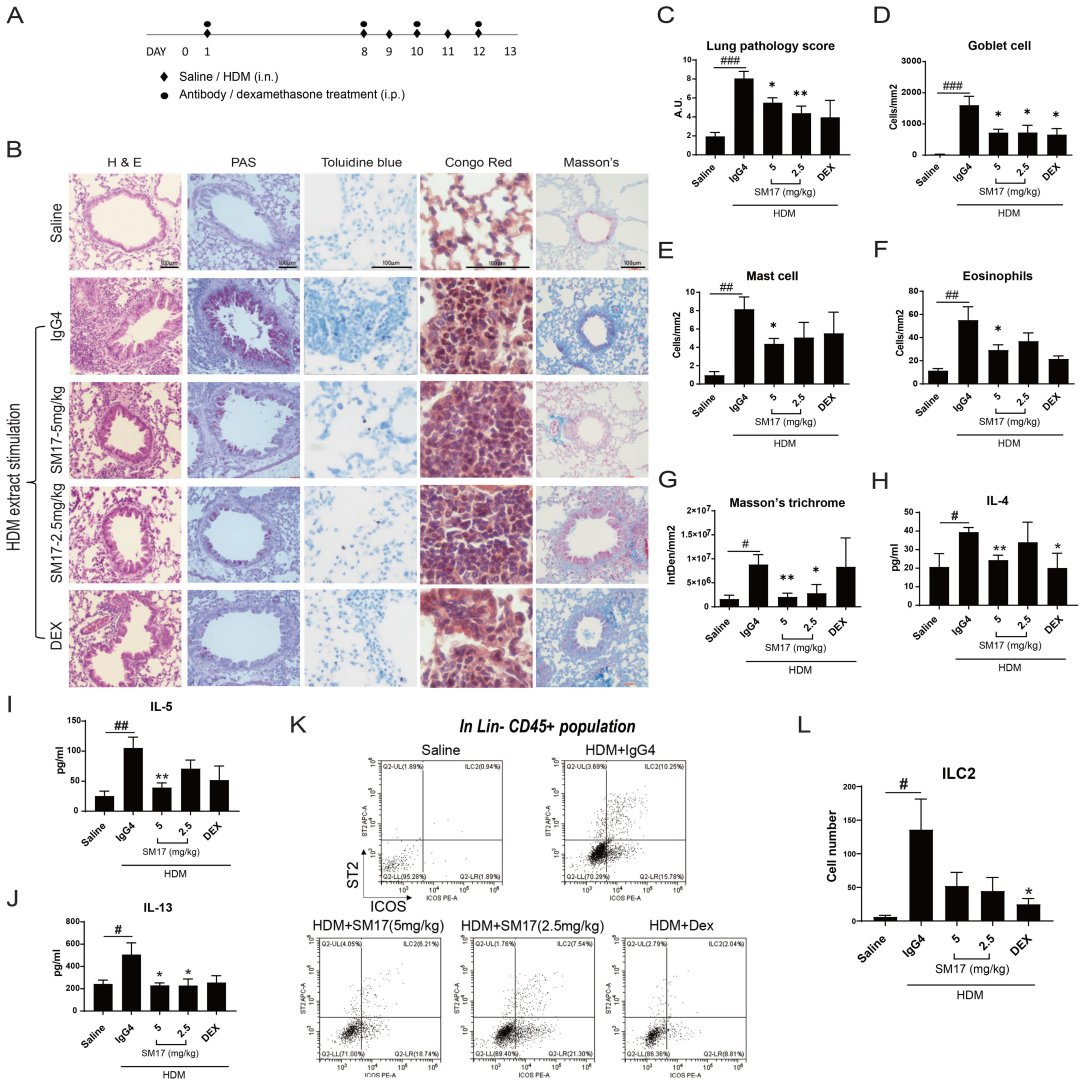
SM17 Abrogated Disease Phenotypes in HDM Extract-Induced Asthma Model. **(A)** Experiment timeline for HDM extract-induced model were shown. **(B)** H&E, PAS, toluidine blue, congo red and Masson’s trichrome staining were performed in paraffin-embedded lung sections. **(C)** SM17 significantly improved lung pathology as stimulated by HDM extract. **(D–F)** Goblet cells, mast cells and eosinophils in lung were all significantly reduced by SM17. **(G)** Masson’s trichome stains for collagen deposition and SM17 exhibited strong inhibitory effects on it, while dexamethasone did not. **(H–J)** Levels of IL-4, IL-5 and IL-13 in BALF were determined by ELISA. SM17 showed similar suppression as dexamethasone on all three Th2 cytokines. **(K, L)** Infiltration of ILC2 in BALF was analysed by flow cytometry as identified by the population Lineage CD45^+^ ICOS^+^ ST2^+^. Results indicated that SM17 possessed high suppressive effects on lung ILC2 numbers. Scale bar = 100μm. *P<.05; **P<.01 compared to IgG4 control group by unpaired student t-test. #P<.05; ##P<0.01; ###P<0.005 as compared to saline control by unpaired student t-test. DEX, dexamethasone; HDM, house dust mite.

Pre-clinical efficacy of SM17 was evaluated by a well-established HDM extract-induced asthma model in mice. A 13-day protocol was designed as presented in **Figure 2A**. Dexamethasone, a corticosteroid drug was applied as positive control. Histological results shown in **Figure 2B**, demonstrated a lower lung pathology score for SM17-treated mice as compared with the IgG4 control group (**Figure 2C**), in which the bronchiole lining was thinner and fewer infiltrated cells were observed. Goblet cells, mast cells and eosinophils were visualized by PAS, toluidine blue and congo red staining, respectively. All three types of cells were significantly suppressed by SM17 treatment (**Figures 2D–F**). In addition, SM17 exhibited comparable inhibitory effects as dexamethasone especially on Goblet cell hyperplasia and eosinophil infiltrations. Furthermore, SM17 possessed strong suppressive effects on collagen deposition as revealed by Masson’s trichrome staining, while dexamethasone treatment did not (**Figure 2G**). Cytokine levels in BALF revealed results consistent with histology findings, showing the ability of SM17 to effectively abrogate allergen-induced IL-4, IL-5 and IL-13 levels in BALF (**Figures 2H–J**) and established inhibitory effects were comparable to dexamethasone. In line with this, flow cytometry was performed (**Figure 2K**) and showed that both doses of SM17 strongly downregulated the ILC2 number in BALF (**Figure 2L**), indicating this is one of the major mechanisms of SM17 to drive down type 2 inflammation. Overall, the optimal dose of SM17 was defined as 5 mg/kg.

### 3.2 Clinical studies: participant characteristics

**Figure 3:**
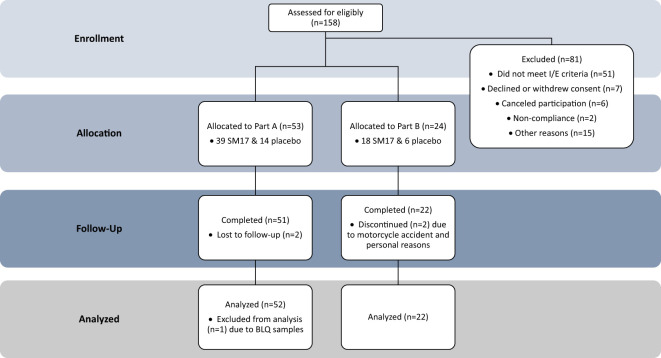
Study Participant Allocation. CONSORT Dose-finding Extension (CONSORT-DEFINE) study participant allocation.

A total of 77 healthy males and females were enrolled in the trial (**Figure 3**). Participant characteristics and disposition are listed in **Table 1**. In Part A, 53 healthy adult subjects were randomized to the study, 39 to active treatment and 14 subjects to placebo. Two subjects did not return for their end of study visit and were considered lost to follow-up. In Part B, 24 subjects were randomized to the study (18 active, 6 placebo). Twenty-two subjects completed the study, while 2 discontinued early, 1 due to a drug-unrelated serious adverse event (SAE) (i.e. motorcycle accident) and another for personal reasons.

**Table 1a:** Participant characteristics (Part A).

Parameters	Part A: Single Ascending Dose									
	2 mgSM17(N = 4)	20 mg SM17 (N = 6)	70 mg SM17 (N = 6)	200 mg SM17 (N = 6)	400 mg SM17 (N = 6)	600 mg SM17 (N = 5)	1200 mg SM17 (N = 6)	Pooled SM17 (N=39)	Pooled Placebo (N = 14)	Total (N=53)
Sex, n	4M	5M/1F	3M/3F	5M/1F	4M/2F	4M/1F	6M	31M/8F	13M/1F	44M/9F
Race, n (%)										
American Indian	0 (0)	1 (17)	0 (0)	0 (0)	0 (0)	0 (0)	0 (0)	1 (3)	0 (0)	1 (2)
Black	0 (0)	1 (17)	0 (0)	1 (17)	0 (0)	1 (20)	3 (50)	6 (15)	2 (14)	8 (15)
White	4 (100)	4 (67)	5 (83)	5 (83)	6 (100)	3 (60)	3 (50)	30 (77)	11 (79)	41 (77)
White, American Indian	0 (0)	0 (0)	0 (0)	0 (0)	0 (0)	1 (20)	0 (0)	1 (3)	0 (0)	1 (2)
White, Asian	0 (0)	0 (0)	1 (17)	0 (0)	0 (0)	0 (0)	0 (0)	1 (3)	0 (0)	1 (2)
White, Black	0 (0)	0 (0)	0 (0)	0 (0)	0 (0)	0 (0)	0 (0)	0 (0)	1 (7)	1 (2)
Ethnicity, n (%)										
Hispanic/Latino	0 (0)	1 (17)	2 (33)	0 (0)	1 (17)	0 (0)	0 (0)	4 (10)	1 (7)	5 (9)
Not Hispanic/Latino	4 (100)	5 (83)	4 (67)	6 (100)	5 (83)	5 (100)	6 (100)	35 (90)	13 (93)	48 (91)
Physical Characteristics, mean ± SD										
Age (years)	42.0 ± 11.4	35.8 ± 10.0	38.5 ± 11.9	36.8 ± 12.1	40.3 ± 11.1	52.8 ± 17.4	41.0 ± 16.2	40.7 ± 13.0	40.6 ± 12.3	40.7 ± 12.8
BMI (kg/m^2^)	23.9 ± 3.9	26.3 ± 3.0	28.3 ± 2.8	25.7 ± 3.0	27.0 ± 4.0	27.5 ± 3.2	28.2 ± 2.0	26.8 ± 3.3	26.9 ± 3.9	26.9 ± 3.5
Weight (kg)	76.8 ± 14.1	82.6 ± 13.3	75.8 ± 8.1	83.7 ± 21.4	85.3 ± 13.4	87.6 ± 8.0	91.2 ± 9.4	83.5 ± 13.3	83.8 ± 10.0	83.6 ± 12.4

**Table 1b:** Participant characteristics Part B.

Parameters	Part B: Multiple Ascending Dose					
	200 mg SM17 Q2W (N = 6)	400 mg SM17 Q2W (N = 6)	600 mg SM17 Q2W (N = 6)	Pooled SM17 Q2W (N=18)	Pooled Placebo Q2W (N = 6)	Total (N=24)
Sex, n	4M/2F	6M	4M/2F	14M/4F	1M/5F	15M/9F
Race, n (%)						
Black	0 (0)	0 (0)	2 (33)	2 (11)	0 (0)	2 (8)
Pacific Islander, Asian	0 (0)	1 (17)	0 (0)	1 (6)	0 (0)	1 (4)
White	6 (100)	4 (67)	4 (67)	14 (78)	6 (100)	20 (83)
White, Black	0 (0)	1 (17)	0 (0)	1 (6)	0 (0)	1 (4)
Ethnicity, n (%)						
Hispanic/Latino	0 (0)	0 (0)	2 (33)	2 (11)	2 (33)	4 (17)
Not Hispanic/Latino	6 (100)	6 (100)	4 (67)	16 (89)	4 (67)	20 (83)
Physical Characteristics, mean ± SD						
Age (years)	49.3 ± 10.0	45.2 ± 16.3	38.8 ± 14.6	44.4 ± 13.8	45.5 ± 14.8	44.7 ± 13.7
BMI (kg/m^2^)	26.6 ± 2.5	27.3 ± 1.9	25.9 ± 3.2	26.6 ± 2.5	27.9 ± 3.8	26.9 ± 2.7
Weight (kg)	79.8 ± 15.4	90.9 ± 11.7	75.0 ± 14.0	81.9 ± 14.7	73.6 ± 11.4	79.8 ± 14.2

BMI, body mass index; F, female; M, male; Q2W, twice weekly.

### 3.3 Safety and tolerability

Overall, TEAEs were observed in 11 (28%) of participants administered SM17 and 4 (29%) dosed with placebo in Part A (**Table 2**). Of the 26 TEAEs reported, the majority (88%) were mild in severity. One moderate TEAE of diarrhea in the 70 mg SM17 cohort was deemed unrelated to study drug, as were 2 severe TEAEs of foot deformity (bunion, 400 mg SM17) and chlamydia (600 mg SM17). The most frequently reported drug-related TEAE was headache.

**Table 2:** Summary of TEAEs.

TEAE Terms	Part A: Single Ascending Dose										Part B: Multiple Ascending Dose					
	2 mg SM17 (N = 4)	20 mg SM17 (N = 6)	70 mg SM17 (N = 6)	200 mg SM17 (N = 6)	400 mg SM17 (N = 6)	600 mg SM17 (N = 5)	1200 mg SM17 (N = 6)	Pooled SM17 (N=39)	Pooled Placebo (N = 14)	Total (N=53)	200 mg SM17 Q2W (N = 6)	400 mg SM17 Q2W (N = 6)	600 mg SM17 Q2W (N = 6)	Pooled SM17 Q2W (N=18)	Pooled Placebo Q2W (N = 6)	Total (N=24)
Number (%) of Subjects with TEAEs																
All TEAEs	1 (25)	1 (17)	4 (67)	2 (33)	2 (33)	1 (20)	0 (0)	11 (28)	4 (29)	15 (28)	4 (67)	4 (67)	3 (50)	11 (61)	5 (83)	16 (67)
Frequency of TEAE Severity																
All TEAEs	1	1	9	5	4	1	0	21	5	26	12	14	8	34	21	55
Severity of TEAEs																
Grade 1	1	1	8	5	3	0	0	18	5	23	10	9	8	27	19	46
Grade 2	0	0	1	0	0	0	0	1	0	1	2	3	0	5	2	2
Grade ≥3	0	0	0	0	1	1	0	2	0	2	0	2 ^a^	0	2 ^a^	0	2 ^a^
Number of TEAEs Related to Study Product																
Decreased appetite	0	0	0	0	0	0	0	0	0	0	2	0	0	2	0	2
Dysphonia	0	0	0	0	0	0	0	0	0	0	1	0	0	1	0	1
Dizziness	0	0	0	1	0	0	0	1	0	1	0	0	0	0	0	0
Feeling hot	0	0	0	0	0	0	0	0	0	0	1	0	0	1	0	1
Headache	0	0	0	1	1	0	0	2	0	2	1	7	1	9	3	12
Tremor	0	0	0	1	0	0	0	1	0	1	0	0	0	0	0	0
Hunger	0	0	0	0	0	0	0	0	0	0	0	1	1	2	0	2
Increased appetite	0	0	0	0	0	0	0	0	0	0	1	0	0	1	0	1
Nausea	0	0	0	0	0	0	0	0	0	0	1	0	0	1	2	3
Peripheral swelling	0	0	0	0	0	0	0	0	0	0	0	0	0	0	1	1
Vomiting	0	0	0	0	0	0	0	0	0	0	0	0	0	0	1	1
**Total:**	**0**	**0**	**0**	**3**	**1**	**0**	**0**	**4**	**0**	**4**	**7**	**8**	**2**	**17**	**7**	**24**

Grade 1 = Mild; Grade 2 = Moderate; Grade 3 = Severe; Grade 4 = Potentially life-threatening. a) Serious adverse event (SAE) was reported related to motorcycle accident, and was not associated with the drug product. Q2W, every two weeks; TEAE, treatment-emergent adverse events.

In Part B, there were 11 (61%) and 5 (83%) participants with TEAEs in the active and placebo groups, respectively (**Table 2**). Thirty-four TEAEs were reported in the active cohorts, in which 17 were considered related to study treatment and the majority were mild in severity. Headache was the most common drug-related TEAEs (50%), observed across all treatment cohorts, and was rated mild to moderate in severity. Changes in appetite or satiety were also relatively common (21%), yet mild in severity. Nausea was the most common TEAE in the placebo group. No severe drug-related TEAEs were reported, however two SAEs (Grade 4), potentially life-threatening TEAEs, were associated with a motorcycle accident (clavicle and wrist fracture), and were not related to study drug (400 mg SM17).

In both Part A and B, no clinically significant changes in serum chemistry, hematology, coagulation, urinalysis, or liver function were reported. In addition, there were no remarkable trends observed in mean vital sign results or ECG parameters, or in changes from baseline following any of the treatments or placebo. Altogether, among the participants administered SM17 there was no dose-dependent trend in the incidence of TEAEs and drug-related TEAEs, and overall single and multiple ascending IV doses of SM17 were well tolerated by the healthy adult subjects.

### 3.4 Part A: pharmacokinetics following a single IV dose of SM17

**Figure 4:**
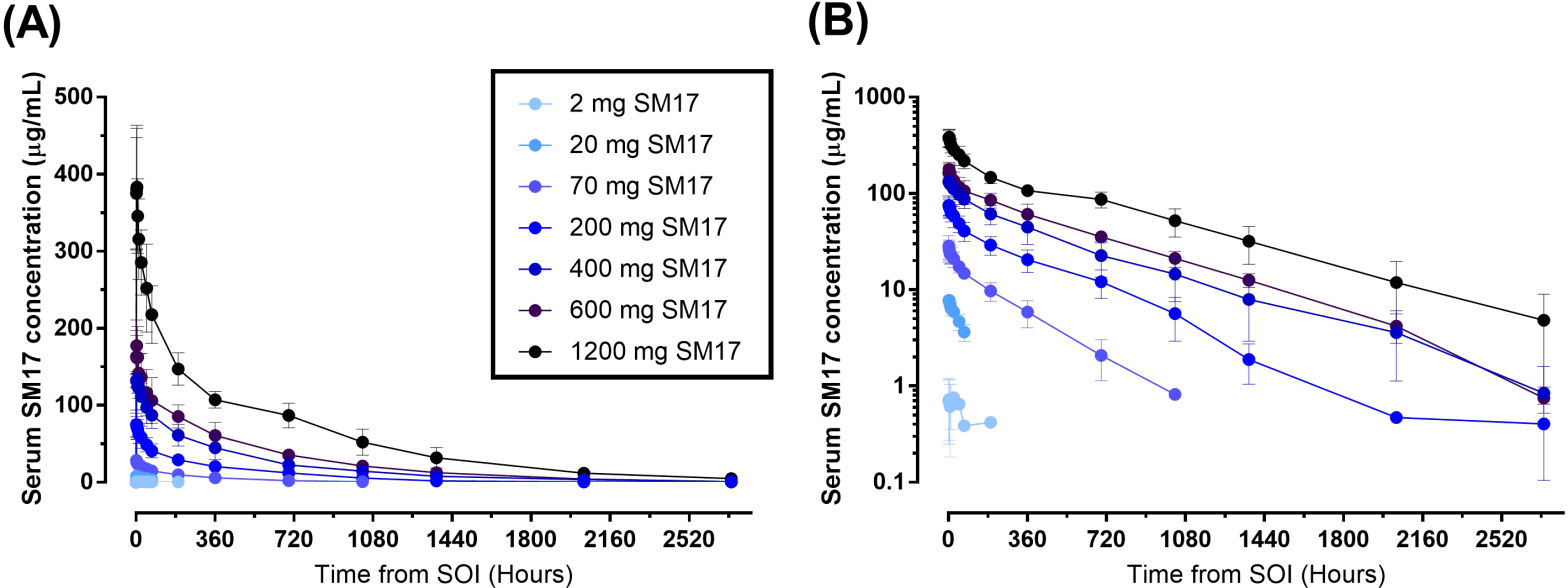
Mean (SD) Serum Concentration-Time Curves after Single Ascending IV Doses of SM17. **(A)** Linear plot; **(B)** Semi-log plot. Abbreviations: **SOI**; start of infusion.

All but one participant was included in the PK analysis set (**Figure 3**). This subject from the 2 mg SM17 group had all their PK sample concentrations below the level of quantification and, thus, was excluded from the analysis. PK parameters following a single IV dose of SM17 are listed in **Table 3**. In Part A, the mean concentration–time profiles showed serum SM17 concentrations increased as dose increased and declined sharply after the infusion after 72 to 168 hours, followed by a more gradual decrease until the end of the sampling interval (**Figure 4**). Peak SM17 exposure (C_max_) increased 616-fold across the explored dose levels of 2 mg to 1200 mg SM17 (**Table 3**). The median T_max_ for SM17 (2.5 to 4 hours) was generally comparable across the dose range. The elimination half-life (t_½_) appeared to increase with increasing dose levels; mean t_½_ values were approximately 83.6 and 184 hours at 20 mg and 70 mg SM17, respectively, and ranged between 294 to 452 hours from 200 mg to 1200 mg. This trend is likely due to SM17 being quantifiable in the majority of subjects over a longer sampling interval as dose increased. Total SM17 exposure, as measured by geometric mean AUCs, increased by approximately 60- to 188-fold with a 60-fold increase in dose from 20 mg to 1200 mg SM17. Mean CL decreased from 20 mg to 200 mg, and was generally comparable at subsequent doses of 200 mg up to 1200 mg. Mean V_z_ steadily increased up to 600 mg SM17, with no further change between 600 mg and 1200 mg SM17 (**Table 3**).

**Table 3:** PK Parameters Following a Single IV Dose of SM17 (Part A and Part B, Day 1).

PK Parameters	Part A: Single Ascending Dose							Part B: Multiple Ascending Dose (Day 1)			
	2 mg SM17 (N=3)	20 mg SM17 (N=6)	70 mg SM17 (N=6)	200 mg SM17 (N=6)	400 mg SM17 (N=6)	600 mg SM17 (N=5)	1200 mg SM17 (N=6)	200 mg SM17 Q2W (N=6)	400 mg SM17 Q2W (N=6)	600 mg SM17 Q2W(N=6)	Dose-Proportionality (Estimate of slope, 95% CI)
C_max_ (μg/mL)	0.6381 (73.2)	7.889 (11.9)	29.65 (26.7)	75.84 (22.9)	134.3 (6.0)	171.0 (23.4)	393.2 (22.0)	75.73 (13.7)	119.6 (15.5)	208.5 (15.1)	0.9694, 0.9295 - 1.0092
T_max_ (hr)	4.001 (3.00, 4.00)	3.038 (2.01, 8.04)	2.508 (2.01, 12.00)	2.513 (2.01, 4.00)	2.510 (2.01, 4.00)	3.000 (2.04, 8.00)	3.009 (2.00, 4.05)	2.016 (2.01, 4.00)	3.520 (2.01, 4.01)	3.004 (2.00, 4.00)	ND
AUC_0-14d_ (μg*hr/mL)	-	776.8 (19.8)	3810 (19.0)	11160 (21.7)	23100 (21.0)	30040 (21.4)	58810 (14.6)	10180 (25.1)	18740 (15.0)	35710 (12.4)	1.0535, 1.0053 - 1.1017
AUC_0-t_ (μg*hr/mL)	10.46 (646.3)	728.1 (26.2)	4982 (32.5)	20520 (29.4)	46000 (37.4)	67590 (17.8)	136600 (23.5)	10170 (25.1)	18720 (15.0)	35680 (12.4)	1.3680, 1.2650 - 1.4711
AUC_0-inf_ (μg*hr/mL)	-	828.9 (23.8)	5422 (29.9)	21160 (29.1)	47200 (36.8)	68870 (18.3)	148600 (21.6) ^a^	16630 (28.7)	32560 (23.1)	74160 (25.8)	1.2579, 1.1901 - 1.3257
t_½_ (hr)	-	83.590 ± 21.1302	184.159 ± 41.0400	294.128 ± 61.7594	352.448 ± 105.3496	395.991 ± 21.7649	452.028 ± 126.3207 ^a^	252.430 ± 18.9647	273.283 ± 36.1479	362.478 ± 90.6066	ND
CL (mL/hr)	-	24.68 ± 5.5797	13.41 ± 4.3680	9.781 ± 2.8394	8.935 ± 3.2032	8.825 ± 1.5600	8.220 ± 1.6729^a^	12.43 ± 3.5038	12.57 ± 3.0646	8.298 ± 1.9487	ND
V_z_ or V_ss_ (L) ^b^	-	2.858 ± 0.35267	3.365 ± 0.38441	3.983 ± 0.74832	4.199 ± 0.97902	5.049 ± 0.99656	5.148 ± 0.82731^a^	4.286 ± 0.92382	4.721 ± 0.46292	4.095 ± 0.44850	ND

AUCs and C_max_ are presented as geometric mean (geometric CV%). T_max_ are presented as median (min, max). Other parameters are presented as arithmetic mean ± SD. ^a^) N=5, ^b^) V_z_ values are presented for Part A and V_ss_ values are presented for Part B. AUC, area-under the curve; CL, clearance; CI, confidence interval; C_max_, maximum concentration; ND, not determined; Q2W, every two weeks; t_½_, half-life; T_max_, time to maximum concentration; V_ss_, volume of distribution at steady state after IV administration; V_z_, volume of distribution during the terminal elimination phase after IV administration.

Dose-proportionality was analyzed for all participants after a single IV administration of SM17 (Part A and B, Day 1). The 95% CIs for the estimated slope for AUC_0-14d_, AUC_0-t_ and AUC_0-inf_ were greater than 1 and, thus, the increase was considered greater than dose-proportional from 2 mg to 1200 mg SM17 (**Table 3**). For C_max_, the 95% CIs of the slope contained 1, but dose proportionality from 2 mg to 1200 mg SM17 could not be confirmed as the quadratic term was significant (data not shown).

### 3.5 Part B: pharmacokinetics following multiple IV doses of SM17

**Figure 5:**
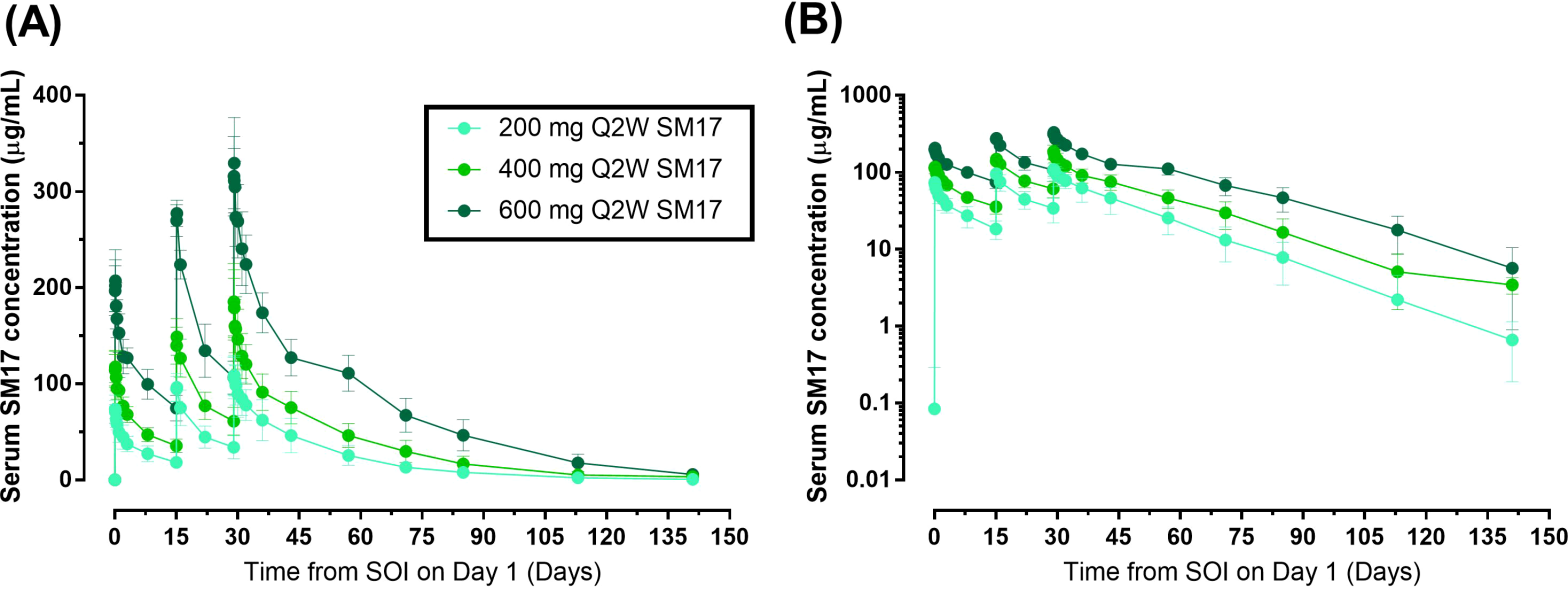
Mean (SD) Serum Concentration-Time Curves after Multiple Ascending IV Doses of SM17. **(A)** Linear plot; **(B)** Semi-log plot. Abbreviations: Q2W, every two weeks; SOI, start of infusion.

In Part B, serum SM17 concentrations increased with increasing doses (**Figure 5**). In addition, drug concentrations within the 2-week dosing interval increased with each additional Q2W administration and peak mean serum SM17 concentrations for each dose were higher after each subsequent administration, suggesting a moderate accumulation of serum SM17. Over the course of the study, SM17 did not reach steady state after three Q2W doses of 200 mg to 600 mg SM17. After administration on Day 29, SM17 AUCs and C_max,ss_ increased by approximately 3-fold in dose from 200 mg to 600 mg while mean CL_ss_ were comparable across the groups (**Table 4**). Mean accumulation ratios over 200-600 mg were 1.5 to 2.1, which confirms moderate accumulation of SM17 between Days 1 and 29 following Q2W administration at each dose level. Moreover, after Q2W dosing of SM17 over 4 weeks, total exposure (AUC_τ_, and AUC_0-t_) increased in a dose-proportional manner from 200 mg to 600 mg SM17 (**Table 4**). Whilst peak exposure (C_max,ss_) increased by a similar proportion to dose, the statistical analysis did not confirm dose linearity of C_max,ss_ across the dose range (data not shown).

**Table 4:** PK Parameters Following Multiple IV Doses of SM17 Q2W (Part B, Day 29).

PK Parameters	200 mg SM17 Q2W (N=6)	400 mg SM17 Q2W (N=5)	600 mg SM17 Q2W (N=6)	Dose-Proportionality (Estimate of slope, 95% CI)
C_max,ss_ (μg/mL)	111.0 (19.7)	185.3 (18.9)	336.1 (12.6)	0.9826, 0.7741 - 1.1910
T_max,ss_ (hr)	3.000 (2.01, 4.01)	2.007 (2.00, 4.01)	3.000 (2.01, 3.19)	ND
AUC_τ_ (μg*hr/mL)	21370 (31.2)	33780 (19.6)	62290 (12.7)	0.9440, 0.6788 - 1.2092
AUC_0-t_ (μg*hr/mL)	44930 (36.9)	81540 (29.8)	167400 (27.1)	1.1653, 0.8118 - 1.5188
t_½_ (hr)	349.933 ± 47.6402	401.185 ± 78.6119	443.387 ± 112.1364	ND
CL_ss_ (mL/hr)	9.735 ± 3.0648	12.02 ± 2.3780	9.695 ± 1.2076	ND
RAAUC_τ_	2.107 ± 0.1857	1.866 ± 0.1958	1.746 ± 0.0669	ND
RAC_max_	1.472 ± 0.1464	1.584 ± 0.1133	1.616 ± 0.1243	ND

AUCs and C_max,ss_ are presented as geometric mean (geometric CV%). T_max,ss_ are presented as median (min, max). Other parameters are presented as arithmetic mean ± SD. AUC, area-under the curve; CL, clearance; CI, confidence interval; C_max_, maximum concentration; ND, not determined; Q2W, every two weeks; t_½_, half-life; RA, accumulation ratio; T_max_, time to maximum concentration.

### 3.6 Immunogenicity

ADAs for SM17 were detected in 8 and 3 participants receiving SM17 in Part A and Part B, respectively. In the single ascending dose cohorts, 2 subjects had positive results prior to SOI. A total of 5 subjects exhibited postdose positive ADA titers on Day 15 (following 20 mg, 70 mg, and 200 mg SM17), 3 subjects on Day 29 (following 20 mg and 200 mg SM17), and 5 subjects on Day 113 (following 70 mg, 200 mg, and 400 mg SM17). In the multiple ascending cohorts, 1 subject had positive results prior to SOI. A total of 2 subjects had positive ADA titers on Day 15 predose (1 subject each in the 200 mg and 600 mg SM17 Q2W treatment groups). Overall, no trend in positive ADA response and dose was observed. The samples were not tested for neutralizing activity.

### 3.7 Eosinophil cell count and lymphocyte phenotyping

**Figure 6:**
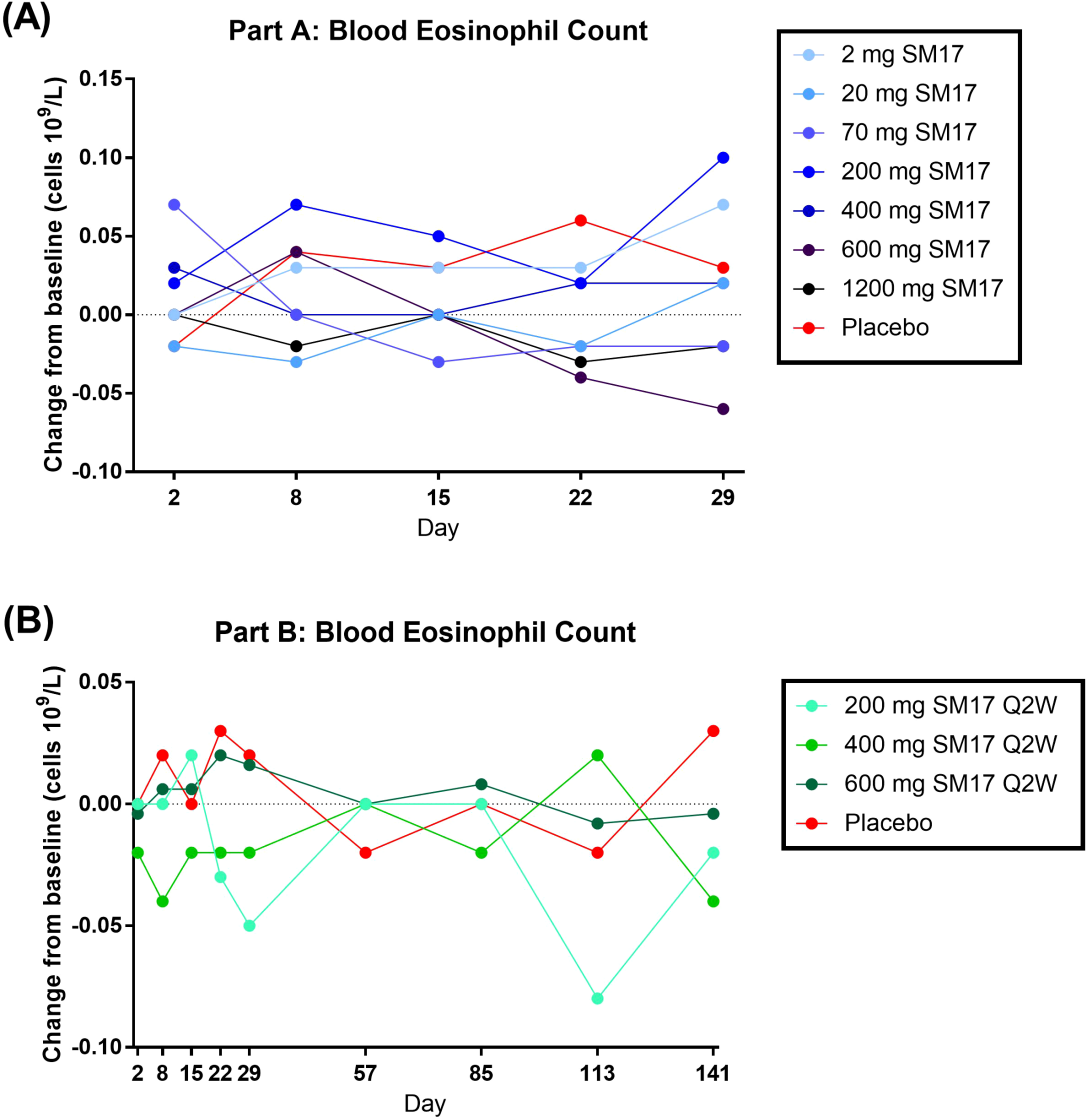
Blood Eosinophil Count Change from Baseline. **(A)** Single Ascending IV Doses of SM17. **(B)** Multiple Ascending IV Doses of SM17. Q2W, every two weeks.

Eosinophil and lymphocyte cell counts and phenotyping were assessed as putative early signals of efficacy, though were expected to minimally change in a healthy adult population. Indeed, the eosinophil cell count change from baseline was negligible upon single (**Figure 6A**) or multiple doses (**Figure 6B**) of SM17. Similarly, little changes in absolute lymphocyte count, %CD4+ and %CD8+ cells were detected across all cohorts (data not shown).

## 4 Discussion

In presenting compelling evidence that SM17 can ameliorate lung histopathology and biomarkers in the current animal model, such as improvements in Th2 cytokines, lung collagen level and cell infiltration, this strengthens the potential of SM17 as an effective treatment for asthma. Together with the promising efficacies in AD (25), SM17 as a first-in-class IL-17RB-targeting monoclonal antibody, was decided to proceed into clinical development. In this Phase I study, SM17 was well tolerated following single IV doses from 2 mg to 1200 mg and multiple IV doses from 200 mg to 600 mg (Q2W) in healthy adults. There were no dose-dependent increases in the incidence of TEAEs. Overall, TEAEs were mild and transient in nature, with headache representing the most common TEAE in both study parts. There were no discontinuations due to drug-related TEAEs, and no deaths were reported. One case of SAE of clavicle and wrist fracture was reported due to motorcycle accident and determined not to be drug-related. In addition, no clinically significant changes from baseline were observed for ECGs, vital signs, physical examinations and laboratory assessments in either active or placebo groups. A few participants had ADAs present both before and after SM17 administration, and no trend in positive ADA was detected with ascending doses of SM17.

Concentration-time profiles from single IV administration of SM17 visually appeared to increase in a dose-dependent manner, however statistical analysis revealed mean AUCs increased in a more than dose-proportional fashion from 2 mg to 1200 mg. On the other hand, total exposure increased dose proportionally from 200 mg to 600 mg over three Q2W administrations. In addition, following multiple ascending doses, moderate accumulation was observed and steady state was not achieved by Day 29. The mean t_½_ of SM17 after 200 – 600 mg multiple doses was approximately 14.6 to 18.5 days. This range is similar to current alarmin inhibitors in development for asthma and AD, such as CNTO 7160, an anti-IL-33 receptor monoclonal antibody (28).

As reported previously, part of the anti-inflammatory effect of attenuating alarmin signaling is a reduction in eosinophils (29–33). Therefore, in the present Phase I trial, eosinophil count was assessed as a putative proof-of-mechanism, however cell levels minimally changed after SM17 administration. This is probably because of the limitation of this study, in which only healthy volunteers were recruited and their basal eosinophil count were already within normal range (0 – 0.5x10^9^/L). In addition, steady-state levels were not obtained after three doses of SM17, which may also contribute to the lack of effect on eosinophils. Even though SM17 administration may not have a significant impact on healthy volunteers, our pre-clinical data suggested that it may possess a more pronounced inhibitory effect on eosinophils in asthmatic subjects. In the physiologically relevant model of allergic asthma, 5 mg/kg dose of SM17 markedly decreased the levels of Th2 cytokines and the number of ILC2 cells in BALF. Additionally, it effectively reduced goblet cell hyperplasia in the bronchial epithelium and the infiltration of mast cells and eosinophils into the lungs, demonstrating strong anti-asthmatic effects in the animals.

Potency of SM17 on suppressing Th2 responses in this asthma model aligns with our previously published findings in a 2,4-dinitrofluorobenzene (DNFB)-induced contact dermatitis model (25). In that study, SM17 also successfully reduced Th2 cytokine levels and epidermal thickness, as well as the infiltrations of mast cells, Th2 cells and eosinophils into the dorsal skin and ear tissues (25). Both models showed SM17 could effectively target multiple disease factors, indicating its potential as a powerful therapeutic option for treating Th2-associated diseases (34).

Many of the approved biologics for severe AD and asthma target ‘downstream’ cytokines in the Th2 inflammation cascade (35–37). For example, dupilumab is a humanized monoclonal antibody targeting alpha chain of IL-4/IL-13 receptor and has been approved for the treatments of both severe AD and asthma (38, 39). IL-5/IL-5R targeting antibodies, including mepolizumab, reslizumab and benralizumab, were also approved for severe eosinophilic asthma unresponsive to other therapies (40, 41). In asthma patients, these ‘downstream’ biologics can only reduce asthma annual exacerbation rates by approximately 50% (40, 42, 43). Therefore, there is still an unmet need for alternative therapies, particularly for patients who do not respond to conventional treatments (44, 45). To that end, therapies targeting ‘upstream’ mediators of the Th2 inflammatory cascade are required to be developed (46, 47). Alarmins such as IL-25, IL-33 and TSLP function at the initial stages of the allergy cascade and are anticipated to have a more comprehensive impact on airway inflammation, potentially offer more effective asthma management than currently therapies (30, 48). Indeed, tezepelumab, a fully humanized IgG2λ monoclonal antibody that specifically inhibits TSLP, was reported to reduce asthmatic exacerbations by 62%-71% (49). It is approved in the US for the add-on maintenance treatment of adult and pediatric patients aged 12 years and older with severe asthma (30). Anti-IL-33/anti-ST2 (IL-33 receptor) monoclonal antibodies also showed promising results in clinical trials (29, 50, 51), illustrating the potential of anti-alarmin therapies for Th2-regulated diseases. Inhibition of the IL-25 pathway is also predicted to have similar clinical outcomes in asthma patients.

**Figure 7:**
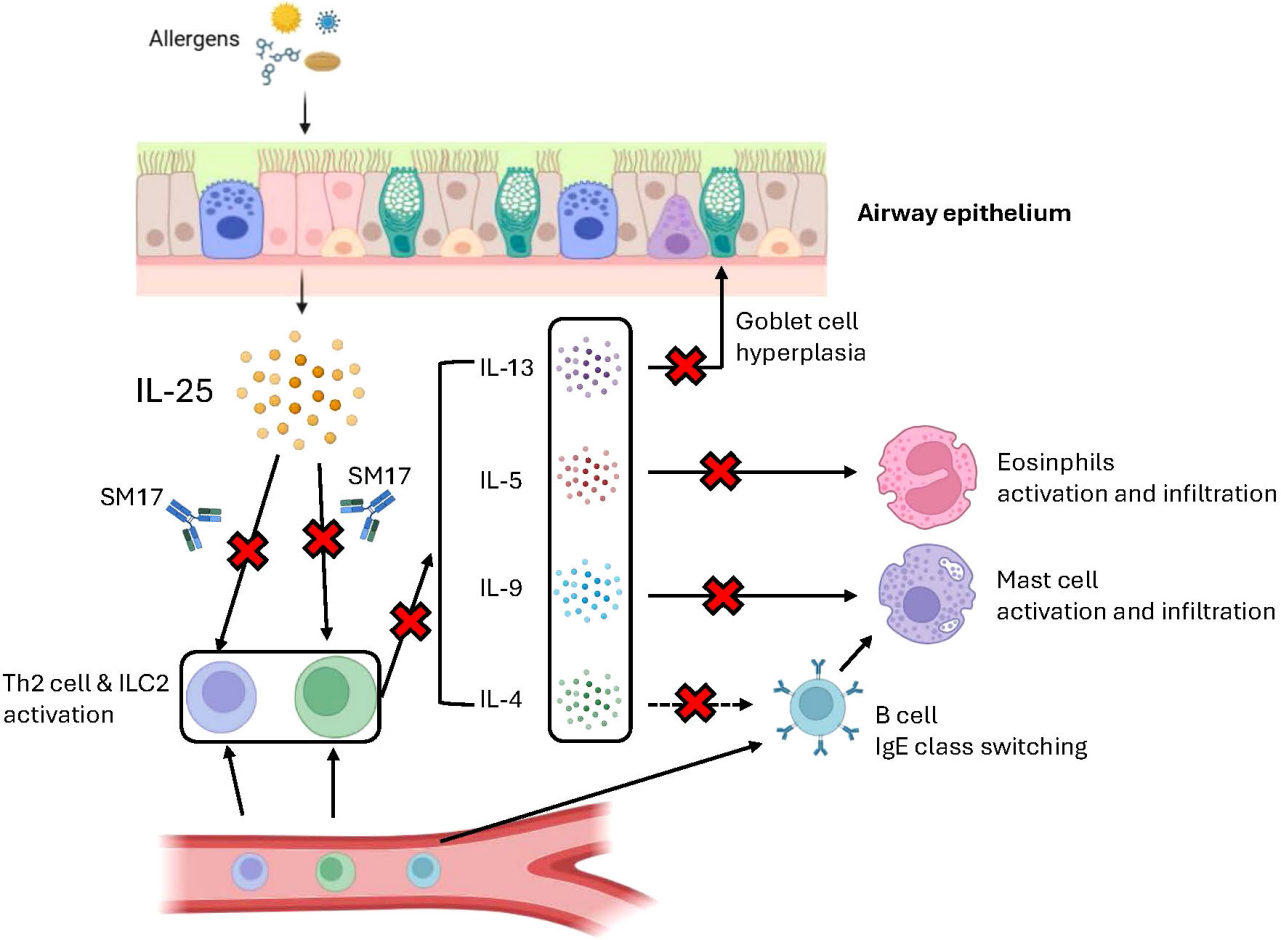
Plausible mechanism of SM17 in asthma. Graphical illustration was created through BioRender.com to explain the mechanism of action of SM17 in asthma.

Based on our pre-clinical studies and several research findings, a graphical illustration was created to explain the plausible mechanisms of SM17 in asthma (**Figure 7**). In brief, when allergens like HDM encounter the airway epithelium, epithelial cells will release IL-25 into peripheral spaces and activate Th2 and ILC2 cells (32, 52). These target cells are responsible for secreting Th2 cytokines (‘downstream’ factors), including IL-4, IL-5, IL-9 and IL-13 (53). Each cytokine has its crucial role in the asthma pathogenesis. IL-4 is known to activate B cell and induce the IgE class-switching process (54, 55). IL-5 plays a critical role in maturation, activation and migration of eosinophils to the airways, which is also a major stimulator of eosinophilia in other Th2-driven diseases (56, 57). IL-9 mainly promotes mast cell proliferation and migration, resulting in mast cell infiltration in allergic airways (58, 59). IL-13 contributes to goblet cell hyperplasia and mucus secretion during airway inflammation, which eventually increases airway resistance and lead to asthma exacerbations (60, 61). As mentioned, IL-25 serves as ‘upstream’ cytokine and contributes to all four Th2 cytokine productions. Blocking of its receptor IL-17RB with SM17 may just offer inhibitory effects on these disease-contributing cytokines all at once (62), which could result in better restoration in normal lung histology and reduction in asthma exacerbations.

Comparing with other IV administrated monoclonal antibody therapies, one advantage SM17 holds is a fixed dose approach to drug administration. While IV treatments such as reslizumab apply weight-based dosing (mg/kg), SM17 was administered as a fixed dose (mg) in this study. As monoclonal antibodies tend to possess a wide therapeutic window, Hendrikx et al. advocate for fixed-dosing schedules since body weight has minor effects on IgG distribution and elimination (63). The authors also suggest that cost effectiveness can be gained in the preparation and administration of fixed-dose compared to body-size based dose therapies.

While the present study provides a relative comprehensive characterization of safety and PK profile for SM17 in healthy subjects, there are some limitations to be addressed, for instance, the safety and PK profile had not been explored in patients with asthma. In addition, the efficacy of SM17 in humans remains to be investigated in future clinical studies.

## 5 Conclusion

The ‘upstream’ role of alarmins in Th2 inflammatory disease makes these cytokines and their receptors ideal targets for treating chronic conditions associated with eosinophilia and high Th2 cytokine levels, such as asthma. As IL-25 contributes to the pathology of asthma, blockade of its receptor by SM17 may serve as a promising approach to treat this disease. Overall, SM17 was well tolerated and the findings of this Phase I study support further drug development in patients.

## Data Availability

The datasets presented in this article are not readily available because of privacy or ethical restrictions. Requests to access the datasets should be directed to Shui-on Leung, shawn@sinomab.com.

## References

[1] Ji T Li H. T-helper cells and their cytokines in pathogenesis and treatment of asthma. Front Immunol. (2023) 14:1149203. doi: 10.3389/fimmu.2023.1149203 37377958 PMC10291091

[2] Zhu J. T helper 2 (Th2) cell differentiation, type 2 innate lymphoid cell (ILC2) development and regulation of interleukin-4 (IL-4) and IL-13 production. Cytokine. (2015) 75:14–24. doi: 10.1016/j.cyto.2015.05.010 26044597 PMC4532589

[3] Fort MM Cheung J Yen D Li J Zurawski SM Lo S. IL-25 induces IL-4, IL-5, and IL-13 and Th2-associated pathologies in vivo. Immunity. (2001) 15:985–95. doi: 10.1016/S1074-7613(01)00243-6 11754819

[4] Hong H Liao S Chen F Yang Q Wang DY. Role of IL-25, IL-33, and TSLP in triggering united airway diseases toward type 2 inflammation. Allergy. (2020) 75:2794–804. doi: 10.1111/all.v75.11 32737888

[5] Harker JA Lloyd CM. T helper 2 cells in asthma. J Exp Med. (2023) 220:e20221094. doi: 10.1084/jem.20221094 37163370 PMC10174188

[6] Rizzi A Di Gioacchino M Gammeri L Inchingolo R Chini R Santilli F. The emerging role of innate lymphoid cells (ILCs) and alarmins in celiac disease: an update on pathophysiological insights, potential use as disease biomarkers, and therapeutic implications. Cells. (2023) 12:1910. doi: 10.3390/cells12141910 37508573 PMC10378400

[7] Cheung PF Wong CK Ip WK Lam CW. IL-25 regulates the expression of adhesion molecules on eosinophils: mechanism of eosinophilia in allergic inflammation. Allergy. (2006) 61:878–85. doi: 10.1111/j.1398-9995.2006.01102.x 16792588

[8] Angkasekwinai P Park H Wang YH Wang YH Chang SH Corry DB. Interleukin 25 promotes the initiation of proallergic type 2 responses. J Exp Med. (2007) 204:1509–17. doi: 10.1084/jem.20061675 PMC2118650 17562814

[9] Global Initiative for Asthma. Global strategy for asthma management and prevention (2024). Available online at: www.ginasthma.org (Accessed November 7, 2024).

[10] Papadopoulos NG Bacharier LB Jackson DJ Deschildre A Phipatanakul W Szefler SJ. Type 2 inflammation and asthma in children: A narrative review. J Allergy Clin Immunol Pract. (2024) 12:2310–24. doi: 10.1016/j.jaip.2024.06.010 38878861

[11] Paplinska-Goryca M Grabczak EM Dabrowska M Hermanowicz-Salamon J Proboszcz M Nejman-Gryz P. Sputum interleukin-25 correlates with asthma severity: a preliminary study. Postepy Dermatol Alergol. (2018) 35:462–9. doi: 10.5114/ada.2017.71428 PMC6232538 30429702

[12] Beale J Jayaraman A Jackson DJ Macintyre JDR Edwards MR Walton RP. Rhinovirus-induced IL-25 in asthma exacerbation drives type 2 immunity and allergic pulmonary inflammation. Sci Transl Med. (2014) 6:256ra134. doi: 10.1126/scitranslmed.3009124 PMC4246061 25273095

[13] Tang W Smith SG Beaudin S Dua B Howie K Gauvreau G. IL-25 and IL-25 receptor expression on eosinophils from subjects with allergic asthma. Int Arch Allergy Immunol. (2014) 163:5–10. doi: 10.1159/000355331 24247484

[14] Corrigan CJ Wang W Meng Q Fang C Eid G Caballero MR. Allergen-induced expression of IL-25 and IL-25 receptor in atopic asthmatic airways and late-phase cutaneous responses. J Allergy Clin Immunol. (2011) 128:116–24. doi: 10.1016/j.jaci.2011.03.043 21570719

[15] Hunninghake GM Chu JH Sharma SS Cho MH Himes BE Rogers AJ. The CD4+ T-cell transcriptome and serum IgE in asthma: IL17RB and the role of sex. BMC Pulm Med. (2011) 11:17. doi: 10.1186/1471-2466-11-17 21473777 PMC3080837

[16] Peng B Sun L Zhang M Yan H Shi G Xia Z. Role of IL-25 on eosinophils in the initiation of th2 responses in allergic asthma. Front Immunol. (2022) 13:842500. doi: 10.3389/fimmu.2022.842500 35615348 PMC9125245

[17] Hongjia L Caiqing Z Degan L Fen L Chao W Jinxiang W. IL-25 promotes Th2 immunity responses in airway inflammation of asthmatic mice via activation of dendritic cells. Inflammation. (2014) 37:1070–7. doi: 10.1007/s10753-014-9830-4 24487979

[18] Kaiko GE Phipps S Angkasekwinai P Dong C Foster PS. NK cell deficiency predisposes to viral-induced Th2-type allergic inflammation via epithelial-derived IL-25. J Immunol. (2010) 185:4681–90. doi: 10.4049/jimmunol.1001758 20855881

[19] Gregory LG Jones CP Walker SA Sawant D Gowers KH Campbell GA. IL-25 drives remodelling in allergic airways disease induced by house dust mite. Thorax. (2013) 68:82–90. doi: 10.1136/thoraxjnl-2012-202003 23093652 PMC3534261

[20] Ballantyne SJ Barlow JL Jolin HE Nath P Williams AS Chung KF. Blocking IL-25 prevents airway hyperresponsiveness in allergic asthma. J Allergy Clin Immunol. (2007) 120:1324–31. doi: 10.1016/j.jaci.2007.07.051 17889290

[21] Zhang FQ Han XP Zhang F Ma X Xiang D Yang XM. Therapeutic efficacy of a co-blockade of IL-13 and IL-25 on airway inflammation and remodeling in a mouse model of asthma. Int Immunopharmacol. (2017) 46:133–40. doi: 10.1016/j.intimp.2017.03.005 28282577

[22] Tang W Smith SG Du W Gugilla A Du J Oliveria JP. Interleukin-25 and eosinophils progenitor cell mobilization in allergic asthma. Clin Transl Allergy. (2018) 8:5. doi: 10.1186/s13601-018-0190-2 29456832 PMC5809891

[23] Jung JS Park BL Cheong HS Bae JS Kim JH Chang HS. Association of IL-17RB gene polymorphism with asthma. Chest. (2009) 135:1173–80. doi: 10.1378/chest.08-1595 19118269

[24] Yuan Q Peng N Xiao F Shi X Zhu B Rui K. New insights into the function of Interleukin-25 in disease pathogenesis. biomark Res. (2023) 11:36. doi: 10.1186/s40364-023-00474-9 37005677 PMC10068183

[25] Lam LH Li W Wu WC Chow KC Au WYD Xu G. SM17, a new IL-17RB-targeting antibody, ameliorates disease progression in a mouse model of atopic dermatitis. Allergy. (2024) 79(6):1625–8. doi: 10.1111/all.16120 38590291

[26] Tang W Dong M Teng F Cui J Zhu X Wang W. Environmental allergens house dust mite-induced asthma is associated with ferroptosis in the lungs. Exp Ther Med. (2021) 22:1483. doi: 10.3892/etm.2021.10918 34765024 PMC8576623

[27] Van Hoecke L Job ER Saelens X Roose K. Bronchoalveolar lavage of murine lungs to analyze inflammatory cell infiltration. J Vis Exp. (2017) 123:55398. doi: 10.3791/55398 PMC5607888 28518083

[28] Nnane I Frederick B Yao Z Raible D Shu C Badorrek P. The first-in-human study of CNTO 7160, an anti-interleukin-33 receptor monoclonal antibody, in healthy subjects and patients with asthma or atopic dermatitis. Br J Clin Pharmacol. (2020) 86:2507–18. doi: 10.1111/bcp.v86.12 PMC7688540 32415720

[29] Wechsler ME Ruddy MK Pavord ID Israel E Rabe KF Ford LB. Efficacy and safety of itepekimab in patients with moderate-to-severe asthma. N Engl J Med. (2021) 385:1656–68. doi: 10.1056/NEJMoa2024257 34706171

[30] Porsbjerg CM Sverrild A Lloyd CM Menzies-Gow AN Bel EH. Anti-alarmins in asthma: targeting the airway epithelium with next-generation biologics. Eur Respir J. (2020) 56:2000260. doi: 10.1183/13993003.00260-2020 32586879 PMC7676874

[31] Panettieri R Jr. Lugogo N Corren J Ambrose CS. Tezepelumab for severe asthma: one drug targeting multiple disease pathways and patient types. J Asthma Allergy. (2024) 17:219–36. doi: 10.2147/JAA.S342391 PMC10960583 38524099

[32] Kosloski MP Kalliolias GD Xu CR Harel S Lai CH Zheng W. Pharmacokinetics and pharmacodynamics of itepekimab in healthy adults and patients with asthma: Phase I first-in-human and first-in-patient trials. Clin Transl Sci. (2022) 15:384–95. doi: 10.1111/cts.13157 PMC8841494 34523807

[33] Gauvreau GM Bergeron C Boulet LP Cockcroft DW Cote A Davis BE. Sounding the alarmins-The role of alarmin cytokines in asthma. Allergy. (2023) 78:402–17. doi: 10.1111/all.15609 PMC10108333 36463491

[34] Gandhi NA Bennett BL Graham NM Pirozzi G Stahl N Yancopoulos GD. Targeting key proximal drivers of type 2 inflammation in disease. Nat Rev Drug Discovery. (2016) 15:35–50. doi: 10.1038/nrd4624 26471366

[35] Pappa G Sgouros D Theodoropoulos K Kanelleas A Bozi E Gregoriou S. The IL-4/-13 axis and its blocking in the treatment of atopic dermatitis. J Clin Med. (2022) 11:5633. doi: 10.3390/jcm11195633 36233501 PMC9570949

[36] Principe S Porsbjerg C Bolm Ditlev S Kjaersgaard Klein D Golebski K Dyhre-Petersen N. Treating severe asthma: Targeting the IL-5 pathway. Clin Exp Allergy. (2021) 51:992–1005. doi: 10.1111/cea.13885 33887082 PMC8453879

[37] Pelaia C Paoletti G Puggioni F Racca F Pelaia G Canonica GW. Interleukin-5 in the pathophysiology of severe asthma. Front Physiol. (2019) 10:1514. doi: 10.3389/fphys.2019.01514 31920718 PMC6927944

[38] Rathinam KK Abraham JJ Vijayakumar TM. Dupilumab in the treatment of moderate to severe asthma: an evidence-based review. Curr Ther Res Clin Exp. (2019) 91:45–51. doi: 10.1016/j.curtheres.2019.100571 31871508 PMC6911908

[39] Wang M Gao XH Zhang L. A review of dupilumab in the treatment of atopic dermatitis in infants and children. Drug Des Devel Ther. (2024) 18:941–51. doi: 10.2147/DDDT.S457761 PMC10981892 38560522

[40] McGregor MC Krings JG Nair P Castro M. Role of biologics in asthma. Am J Respir Crit Care Med. (2019) 199:433–45. doi: 10.1164/rccm.201810-1944CI PMC6835092 30525902

[41] Alam N Latha S Kumar A. Safety and efficacy of monoclonal antibodies targeting IL-5 in severe eosinophilic asthma: A systematic review and meta-analysis of randomized controlled trials. Health Sci Rev. (2023) 8. doi: 10.1016/j.hsr.2023.100103

[42] Nagase H Tamaoki J Suzuki T Nezu Y Akiyama S Cole AL. Reduction in asthma exacerbation rate after mepolizumab treatment initiation in patients with severe asthma: A real-world database study in Japan. Pulm Pharmacol Ther. (2022) 75:102130. doi: 10.1016/j.pupt.2022.102130 35714883

[43] Busse WW. Biological treatments for severe asthma: A major advance in asthma care. Allergol Int. (2019) 68:158–66. doi: 10.1016/j.alit.2019.01.004 30792118

[44] Menzies-Gow A Wechsler ME Brightling CE. Unmet need in severe, uncontrolled asthma: can anti-TSLP therapy with tezepelumab provide a valuable new treatment option? Respir Res. (2020) 21:268. doi: 10.1186/s12931-020-01505-x 33059715 PMC7560289

[45] Caminati M Vaia R Furci F Guarnieri G Senna G. Uncontrolled asthma: unmet needs in the management of patients. J Asthma Allergy. (2021) 14:457–66. doi: 10.2147/JAA.S260604 PMC8104981 33976555

[46] Chan R Stewart K Misirovs R Lipworth BJ. Targeting downstream type 2 cytokines or upstream epithelial alarmins for severe asthma. J Allergy Clin Immunol Pract. (2022) 10:1497–505. doi: 10.1016/j.jaip.2022.01.040 35131510

[47] Barnes PJ. Targeting cytokines to treat asthma and chronic obstructive pulmonary disease. Nat Rev Immunol. (2018) 18:454–66. doi: 10.1038/s41577-018-0006-6 29626211

[48] Whetstone CE Ranjbar M Omer H Cusack RP Gauvreau GM. The role of airway epithelial cell alarmins in asthma. Cells. (2022) 11:1105. doi: 10.3390/cells11071105 35406669 PMC8997824

[49] Corren J Parnes JR Wang L Mo M Roseti SL Griffiths JM. Tezepelumab in adults with uncontrolled asthma. N Engl J Med. (2017) 377:936–46. doi: 10.1056/NEJMoa1704064 28877011

[50] Kelsen SG Agache IO Soong W Israel E Chupp GL Cheung DS. Astegolimab (anti-ST2) efficacy and safety in adults with severe asthma: A randomized clinical trial. J Allergy Clin Immunol. (2021) 148:790–8. doi: 10.1016/j.jaci.2021.03.044 33872652

[51] Chinthrajah S Cao S Liu C Lyu SC Sindher SB Long A. Phase 2a randomized, placebo-controlled study of anti-IL-33 in peanut allergy. JCI Insight. (2019) 4:e131347. doi: 10.1172/jci.insight.131347 31723064 PMC6948865

[52] Claudio E Wang H Kamenyeva O Tang W Ha HL Siebenlist U. IL-25 orchestrates activation of th cells via conventional dendritic cells in tissue to exacerbate chronic house dust mite-induced asthma pathology. J Immunol. (2019) 203:2319–27. doi: 10.4049/jimmunol.1900254 PMC6783368 31511356

[53] Duchesne M Okoye I Lacy P. Epithelial cell alarmin cytokines: Frontline mediators of the asthma inflammatory response. Front Immunol. (2022) 13:975914. doi: 10.3389/fimmu.2022.975914 36311787 PMC9616080

[54] Keegan AD Leonard WJ Zhu J. Recent advances in understanding the role of IL-4 signaling. Fac Rev. (2021) 10:71. doi: 10.12703/r/10-71 34557875 PMC8442009

[55] Laidlaw BJ Cyster JG. Transcriptional regulation of memory B cell differentiation. Nat Rev Immunol. (2021) 21:209–20. doi: 10.1038/s41577-020-00446-2 PMC7538181 33024284

[56] Nagase H Ueki S Fujieda S. The roles of IL-5 and anti-IL-5 treatment in eosinophilic diseases: Asthma, eosinophilic granulomatosis with polyangiitis, and eosinophilic chronic rhinosinusitis. Allergol Int. (2020) 69:178–86. doi: 10.1016/j.alit.2020.02.002 32139163

[57] Gevaert P Han JK Smith SG Sousa AR Howarth PH Yancey SW. The roles of eosinophils and interleukin-5 in the pathophysiology of chronic rhinosinusitis with nasal polyps. Int Forum Allergy Rhinol. (2022) 12:1413–23. doi: 10.1002/alr.v12.11 PMC9790271 35243803

[58] Pajulas A Fu Y Cheung CCL Chu M Cannon A Alakhras N. Interleukin-9 promotes mast cell progenitor proliferation and CCR2-dependent mast cell migration in allergic airway inflammation. Mucosal Immunol. (2023) 16:432–45. doi: 10.1016/j.mucimm.2023.05.002 PMC10482122 37172907

[59] Reitz M Hartmann W Rudiger N Orinska Z Brunn ML Breloer M. Interleukin-9 promotes early mast cell-mediated expulsion of Strongyloides ratti but is dispensable for generation of protective memory. Sci Rep. (2018) 8:8636. doi: 10.1038/s41598-018-26907-2 29872093 PMC5988711

[60] Seibold MA. Interleukin-13 stimulation reveals the cellular and functional plasticity of the airway epithelium. Ann Am Thorac Soc. (2018) 15:S98–S102. doi: 10.1513/AnnalsATS.201711-868MG 29676620 PMC5955044

[61] Tukler Henriksson J Coursey TG Corry DB De Paiva CS Pflugfelder SC. IL-13 stimulates proliferation and expression of mucin and immunomodulatory genes in cultured conjunctival goblet cells. Invest Ophthalmol Vis Sci. (2015) 56:4186–97. doi: 10.1167/iovs.14-15496 PMC4495812 26132778

[62] Petersen BC Dolgachev V Rasky A Lukacs NW. IL-17E (IL-25) and IL-17RB promote respiratory syncytial virus-induced pulmonary disease. J Leukoc Biol. (2014) 95:809–15. doi: 10.1189/jlb.0913482 PMC3984969 24407884

[63] Hendrikx J Haanen J Voest EE Schellens JHM Huitema ADR Beijnen JH. Fixed dosing of monoclonal antibodies in oncology. Oncologist. (2017) 22:1212–21. doi: 10.1634/theoncologist.2017-0167 PMC5634778 28754722

