# Supplementary material for: Evaluation of the safety, tolerability, pharmacokinetics and pharmacodynamics of SM17 in healthy volunteers: results from pre-clinical models and a first-in-human, randomized, double blinded clinical trial
Source: Front Immunol. 2024 Dec 9;15:1495540. doi: 10.3389/fimmu.2024.1495540 (PMC11663749; doi:10.3389/fimmu.2024.1495540)
Supplement: Supplementary file 1 [file Table1.docx]

Clinical Synopsis

A Phase 1, First-in-Human, Double-Blind, Placebo-Controlled Study to Investigate the Safety, Tolerability, Pharmacokinetics, and Pharmacodynamics of SM17 when Administered Intravenously as a Single Ascending Dose (Part A) and as Multiple Ascending Doses (Part B) in Healthy Subjects

| Compound: | SM17 |
| --- | --- |
| Clinical Indication: | Treatment of patients with asthma |
| Study Phase and Type: | Phase 1 – single ascending dose (SAD) and multiple ascending doses (MAD) first-in-human (FIH) study |
| Study Objectives: | **Part A (SAD):**  **Primary Objective:**   - To evaluate the safety and tolerability of single intravenous (IV) doses of SM17 in healthy adult subjects.   **Secondary Objectives:**   - To characterize the pharmacokinetics (PK), pharmacodynamics (PD), and immunogenicity of single IV doses of SM17 in healthy adult subjects.   **Exploratory Objectives:**   - To explore the relationship between dose level, exposure level, and safety/tolerability findings in healthy adult subjects.   **Part B (MAD):**  **Primary Objective:**   - To evaluate the safety and tolerability of multiple IV doses of SM17 in healthy adult subjects.   **Secondary Objective:**   - To characterize the PK, PD, and immunogenicity of multiple IV doses of SM17 in healthy adult subjects.   **Exploratory Objectives:**   - To explore the relationship between dose level, exposure level, and safety/tolerability findings in healthy adult subjects. |
| Summary of Study Design: | This is a 2-part, FIH, randomized, double-blind, placebo-controlled study to evaluate the safety, PK, PD, and immunogenicity of SM17 following single ascending IV infusion (Part A) and multiple ascending IV infusion (Part B) in healthy adult subjects.  The details of each study part are as follows:  **Part A (SAD):**  Part A will be conducted in up to 6 SAD cohorts with the possibility of conducting an additional cohort. The starting dose level (Cohort A1) will be the first cohort to be conducted and will enroll approximately 6 subjects (4 SM17 and 2 placebo). The remaining cohorts will enroll approximately 8 subjects each (6 SM17 and 2 placebo).  In each cohort, a sentinel group (1 SM17 and 1 placebo) which will be dosed at least 48 hours before the remaining subjects (4 subjects in Cohort A1 [3 SM17 and 1 placebo] and 6 subjects in the remaining cohorts [5 SM17 and 1 placebo]). Dosing of the remaining subjects will be conducted following a safety evaluation of the sentinel group by the safety review committee (SRC).  In each cohort, subjects will receive a single IV infusion of SM17 or placebo.  Safety (i.e., adverse events [AEs], 12-lead safety electrocardiograms [ECGs], vital signs, clinical laboratory tests, immunophenotyping, infusion site reaction, and physical examinations) will be assessed throughout the study. Immunogenicity (anti-drug antibody [ADA]) will be included in the safety monitoring when results will be available.  Blood samples will be collected for the PK (free SM17) and PD (eosinophil [EOS] count) assessments of SM17 prior to the start of infusion (SOI) and for up to Day 113 (± 7 days).  Dose escalation to the next dose level (i.e., next cohort) will not take place until the SRC has determined that adequate safety and tolerability have been demonstrated in previous cohort(s) to permit proceeding to the next cohort.  Interim PK and PD analyses may be performed to reconsider the sampling time points and for dose-escalation decisions as Part A progresses.  **Part B (MAD):**  Part B will be initiated after completion of the review by the SRC of all pertinent blinded safety and tolerability data for all subjects who completed scheduled study procedures of at least Day 15 (± 1 day) following SM17/placebo dosing in Cohort A5 and for all previous cohorts in Part A. Interim PK and PD analyses from cohorts in Part A will be performed to reconsider the sampling time points, dose levels, and regimen selections.  Part B will be conducted in up to 3 MAD cohorts of approximately 8 subjects each (6 SM17 and 2 placebo) with the lowest dose level (Cohort B1) to be conducted first.  In each cohort, a sentinel group (1 SM17 and 1 placebo) will receive at least the second dose scheduled on Day 15 (± 1) and must complete through at least all of Day 16 (± 1 day) scheduled study procedures prior to the first dosing of the remaining 6 subjects (5 SM17 and 1 placebo). Dosing of the remaining 6 subjects will be conducted following a safety evaluation of the sentinel group by the SRC.  In each cohort, subjects will receive a single IV infusion of SM17 or placebo every 2 weeks (Q2W) over a period of 4 weeks (3 doses total).  Safety (i.e., AEs, 12-lead safety ECGs, vital signs, clinical laboratory tests, immunophenotyping, infusion site reaction, and physical examinations) will be assessed throughout the study. Immunogenicity (ADA]) will be included in the safety monitoring when results will be available.  Blood samples will be collected for the PK (free SM17) and PD (EOS count) assessments of SM17 prior to SOI on Day 1 and up to Day 8, prior to SOI on Day 15 (± 1 day) and on Day 22 (± 2 days), prior to SOI on Day 29 and for up to Day 141 (± 7 days).  Dose escalation to the next dose level (i.e., next cohort) will not take place until the SRC has determined that adequate safety and tolerability have been demonstrated in previous cohort(s) to permit proceeding to the next cohort.  Interim PK and PD analyses will be performed prior to conducting Cohorts B2 and B3 to reconsider dose levels, dosing regimen, sampling time points, and for dose-escalation decisions.  **All Parts:**  Additional cohorts may be enrolled if it is deemed appropriate to repeat any dose level, or to add an interim dose level(s) (higher or lower than those planned), as determined by the SRC, depending on the safety and tolerability results from the prior cohort(s). PK and PD results may also be reviewed prior to enrolment of additional cohorts.  Subjects who received SM17/placebo dosing and who terminate the study early will be asked to return to the Clinical Research Unit (CRU) 14 and 28 days (± 2 days) after the last dose, as applicable, for follow‑up procedures, sample collection for ADA, and to determine if any AE has occurred since the last study visit. |
| Study Population: | Subjects will be healthy, male and female (of non‑childbearing potential) adult subjects consisting of members of the community at large. |
| Number of Subjects: | Up to 78 healthy adult subjects are planned to be enrolled. Subjects will participate in only 1 study part and 1 cohort.  **Part A (SAD):**  Up to 46 healthy adult subjects are planned to be enrolled in 6 cohorts (6 subjects in Cohort A1 [4 SM17, 2 placebo] and 8 subjects in each of the remaining cohort [6 SM17, 2 placebo]) with the option of an additional cohort of 8 subjects (6 SM17, 2 placebo) to be conducted at the discretion of the Sponsor.  **Part B (MAD):**  Up to 24 healthy adult subjects are planned to be enrolled in 3 cohorts (8 subjects in each cohort; 6 SM17, 2 placebo). |
| Dosage, Dosage Form, Route, and Dose Regimen: | **Part A (SAD):**  In Part A, healthy adult subjects will receive a single IV infusion of SM17 or placebo over a period of 2 hours into a peripheral vein.  Planned doses will be as follows:   \| Cohort A1: \| 2 mg SM17 or matching placebo \| \| --- \| --- \| \| Cohort A2: \| 20 mg SM17 or matching placebo \| \| Cohort A3: \| 70 mg SM17 or matching placebo \| \| Cohort A4: \| 200 mg SM17 or matching placebo \| \| Cohort A5: \| 400 mg SM17 or matching placebo \| \| Cohort A6: \| 600 mg SM17 or matching placebo \| \| Cohort A7 \| 1200 mg SM17 or matching placebo \|   The dose level in each cohort may be adjusted based on emerging data available from previous cohorts in Part A.  **Part B (MAD):**  In Part B, healthy adult subjects will receive a single IV infusion of SM17 or placebo over a period of 2 hours into a peripheral vein Q2W over a period of 4 weeks. In each cohort, subjects will receive a total of 3 doses of SM17 or placebo.  Planned doses will be as follows:   \| Cohort B1: \| 200 mg SM17 or matching placebo \| \| --- \| --- \| \| Cohort B2: \| 400 mg SM17 or matching placebo \| \| Cohort B3: \| 600 mg SM17 or matching placebo \|   After initial morning dosing on Day 1, each subsequent morning dose will be administered within ±1 hour of the dosing time established on Day 1.  Dose level and regimen in each cohort may be adjusted based on emerging data available from Part A and available data from previous cohorts (if any) in Part B.  **All Parts:**  Cohort A1, A2, and A3 doses will be delivered as an infusion of 4, 40, and 140 mL, respectively. The dose in the remaining cohorts (Parts A and B) will be delivered as an infusion of 200 mL. The IV infusion will be performed by programmable infusion pump while subjects are seated or semi‑recumbent in bed.  Dose level and dosing regimen in each cohort may be adjusted based on emerging data from previous cohorts in current study part and data from the previous study part.  The highest dose to be assessed in the study will not exceed 1,200 mg or be predicted to exceed a mean serum AUC_0-168_ of 210,000 µg•hr/mL, based on safety and PK data review from previous cohorts, preclinical PK data and previous feedback from the Food and Drug Administration (FDA). |
| Safety Assessments and Analysis: | Safety will be monitored through AEs, 12‑lead ECGs, vital signs measurements (blood pressure [BP], heart rate [HR], respiratory rate [RR] and temperature [T]), clinical laboratory tests (including, but not limited to, hematology, serum chemistry, coagulation, and urinalysis), immunophenotyping, infusion site reaction, immunogenicity, and physical examinations.  The following analyses will be performed; however no formal inferential statistics will be done on safety assessments.  The placebo subjects from all cohorts in each study part will be pooled into a single placebo group for all summaries and presentations.  Descriptive statistics will be calculated for quantitative safety data and frequency counts will be compiled for classification of qualitative safety data for each study part.  **Adverse Events:**  AEs will be coded using the most current version of Medical Dictionary for Regulatory Activities^®^ (MedDRA^®^) available.  A by-subject AE data listing, including verbatim term, preferred term, treatment, severity, and relationship to drug, will be provided.  The number of subjects experiencing treatment-emergent adverse events (TEAEs) and number of TEAEs will be summarized by treatment using frequency counts.  Infusion site reaction will be assessed.  **Medical History:**  Medical history will be listed by subject.  **Clinical Laboratory Results, Immunophenotyping, Electrocardiograms, and Vital Signs Measurements:**  All clinical laboratory and immunophenotyping (including CD4+/CD8+) results, 12-lead ECGs, vital signs measurements, and their change from baseline, will be summarized by treatment and time point of collection.  A shift table describing out-of-normal range shifts will be provided for clinical laboratory results.  **Concomitant Medications:**  Concomitant medications will be coded using the most current World Health Organization (WHO) drug dictionary available and listed by treatment.  **Immunogenicity:**  ADA detection will be reported and summarized descriptively. |
| Pharmacokinetic Assessments and Analyses: | The following non‑compartmental PK parameters for free SM17 will be calculated as appropriate, including but not limited to:  **Part A (SAD):**  AUC_0-t_, AUC_0-24_, AUC_0-inf_, AUC_0-14d_, C_max_, T_max_, t½, CL, and V_z_.  **Part B (MAD):**  AUC_0-24_, AUC_0-14d_, AUC_τ_, C_max_, C_trough_, T_max_, t½, CL, V_ss_, RA_AUC_, and RA_Cmax_.  **All Parts:**  Additional PK parameters may be calculated if deemed appropriate.  Serum PK parameters will be summarized by treatment using descriptive statistics. Linear and semi-logarithmic graphs of mean serum SM17 concentrations by cohort will be drawn.  SM17 dose proportionality may be assessed using the power model approach, as appropriate.  Steady state analyses will be performed in Part B. |
| Pharmacodynamic Assessments and Analysis: | Blood EOS counts will be assessed as a PD biomarker in each part:  The placebo subjects from all cohorts in each study part will be pooled into a single placebo group for all summaries and presentations.  Absolute values and change from baseline values for each PD biomarker will be presented graphically and summarized by treatment using descriptive statistics. |
